# The association of MTHFR C677T variant with increased risk of ischemic stroke in the elderly population: a meta-analysis of observational studies

**DOI:** 10.1186/s12877-019-1304-y

**Published:** 2019-11-27

**Authors:** Guilin Chang, Zheng Kuai, Jia Wang, Jiayu Wu, Kan Xu, Ying Yuan, Yu Hu

**Affiliations:** Department of Geriatrics, Zhongshan Hospital, Fudan University, 180 Fenglin Road, Shanghai, 200032 China

**Keywords:** MTHFR C677T variant, Ischemic stroke, Elderly population, Meta-analysis

## Abstract

**Background:**

C677T point mutation in methylenetetrahydrofolate reductase (MTHFR) gene have been found to be associated with ischemic stroke in general population, while the results seem inconsistent. We aim to assess the association between variant MTHFR C677T variant and increased risk of ischemic stroke and focus on the elderly population.

**Methods:**

We searched PubMed, Embase, Cochrane Library, and Web of Science for eligible studies. Odds ratios (ORs) were calculated with the two-tailed 95% confidence intervals (CIs) by using a random effects model to evaluate any possible association. Among the Chinese and non-Chinese populations, we conducted a subgroup analysis.

**Results:**

The electronic database search yielded 1,358 citations as of December 2017; finally, nine case-control studies involving 3,337 subjects fulfilled our eligibility criteria for inclusion in the study. The pooled results showed that MTHFR C677T variant increased the risk of ischemic stroke (OR = 1.23, 95%CI 1.06–1.43, *P* = 0.0067 for CT + TT vs. CC; OR = 1.18, 95%CI 1.01–1.38, *P* = 0.0333 for CT vs. CC; OR = 1.41, 95%CI 1.14–1.75, *P* = 0.0016 for TT vs. CC; OR = 1.27, 95%CI 1.05–1.54, *P* = 0.0145 for TT vs. CC + CT; OR = 1.18, 95%CI 1.06–1.31, *P* = 0.0023 for T-allele vs. C-allele). Further subgroup analyses in the Chinese population indicated that MTHFR C677T variant was associated with a higher risk of ischemic stroke.

**Conclusion:**

Our findings showed that T-allele increases risk for stroke in the pooled sample. This association was statistically significant in the Chinese cohorts and showed a similar trend in the non-Chinese cohorts. (Word count: 237).

## Background

Stroke is the second leading cause of mortality worldwide and is the most common cause of long-term disability [1]. It can affect individuals of any age. Furthermore, its incidence and prevalence grow with age rapidly [2, 3]. Prior studies indicated that > 70% of deaths due to stroke in China occurred in people aged ≥65 years [4–6]. China has the largest population in the world, which will make its problem of aging society more intractable considering the existing and potentially increasing population of elderly people [7]. Hence, effective solutions are expected.

Ischemic stroke is a clinical condition characterised by reduced blood supply to parts of the human brain. The inadequate blood supply might initiate the ischemic cascade and further lead to dysfunction of the affected parts of brain tissue [8]. Ischemic stroke as a multifactorial disease [9] involving the interaction between many genes and environmental factors [10]. Mutations occurring in the candidate genes have been found to be associated with ischemic stroke [11]. One of the most extensively studied gene variants is on C677T in the methylenetetrahydrofolate reductase (MTHFR) gene such as MTHFR C677T [12].

MTHFR, a folate-dependent enzyme, by converting 5, 10-methylenetetrahydrofolate to 5-methyltetrahydrofolate, plays a critical role in regulating plasma homocysteine levels [13]. A C677T point mutation has been observed to be linked to an increased risk of ischemic stroke in certain population studies [14–16], while inconsistent findings were reported in the general population [17]. Given these findings, we further assessed the association between MTHFR C677T variant and increased risk of ischemic stroke and focus on the elderly population.

## Methods

### Study search

Eligible studies were to be identified by searching electronic databases (PubMed, Embase, Cochrane library, and Web of Science) by using the following search terms: (*‘single nucleotide polymorphism (SNP)’ or ‘SNP’ or ‘mutation’ or ‘genetic polymorphism’ or ‘variation’ or ‘polymorphism’ or ‘variant’*) and (*‘ischemic stroke’ or ‘cerebral ischemic stroke’ or ‘cerebral infarction’*) and (‘*methylenetetrahydrofolate reductase’ or ‘MTHFR’ or ‘methylene tetrahydrofolate reductase’ or ‘5,10-Methylenetetrahydrofolate reductase’*). In addition, the reference lists of original and review articles on the same topic were searched manually.

### Study selection

All the studies were examined thoroughly to assess their appropriateness for inclusion. The following criteria were used to select studies: (1) published as an original article with cohort or case-control study design; (2) a diagnosis of ischemic stroke reported in the case group and absence of this disease in the control group; (3) MTHFR C677T genetic variant status was classified for each patient; (4) the mean (median) ages of the subjects were > 65 years in the study groups; and (5) published in the English language. Studies that provided no information on patients’ average age were excluded from the meta-analysis.

### Data extraction and quality assessment

For the meta-analysis of MTHFR C677T genetic polymorphism and ischemic stroke, the following information was extracted from each study: (1) first author’s name; (2) publication year; (3) study groups and their respective numbers of cases and controls; (4) numbers or percentages of subjects with the CC, CT, and TT genotypes in the cases and controls; and (5) baseline characteristics of the study patients (age, sex, ethnicity, etc).

We used standardised data recording sheets to extract data from the included studies. Two investigators (Z.K. and J.W.) separately performed the screening of study titles and abstracts, full-text reading, and final data extraction. In case of disagreement, the third investigator (JY.W.) was consulted to reach a final agreement. The potential risk for bias was evaluated in accordance with the Preferred Reporting Items for Systematic Reviews and Meta-analysis recommendations [18]. Study quality assessment was performed by using the Newcastle-Ottawa Scale (NOS) [19]. In a meta-analysis, NOS, with the range of 0–9 stars and six stars as high quality, is extensively used for assessing the quality of non-randomized studies.

### Statistical analysis

The meta-analysis was performed using R 3.4.3 (R Foundation for Statistical Computing, Vienna, Austria; http://www.R-project.org/) and the Meta package [20]. We made frequency tables to analyse the potential association between MTHFR C677T variant and the risk of ischemic stroke. With the subject frequency or allele frequency, the odds ratios (ORs) were calculated with the two-tailed 95% confidence intervals (CI). Heterogeneity was defined as an *I*^2^ value of > 50% [21] or *P* value of < 0.10 in the Cochrane Q test [22]. The two indices assess the percentage of variability attributable to heterogeneity across studies rather than by chance. We used random effects model in the meta-analysis. The funnel plots for the ORs were visually inspected and further Egger’s regression test was used to evaluate publication bias statistically [23]. For all the statistical tests, significance was defined as a two-tailed P value of ≤0.05.

## Results

### Search results and study characteristics

**Fig. 1:**
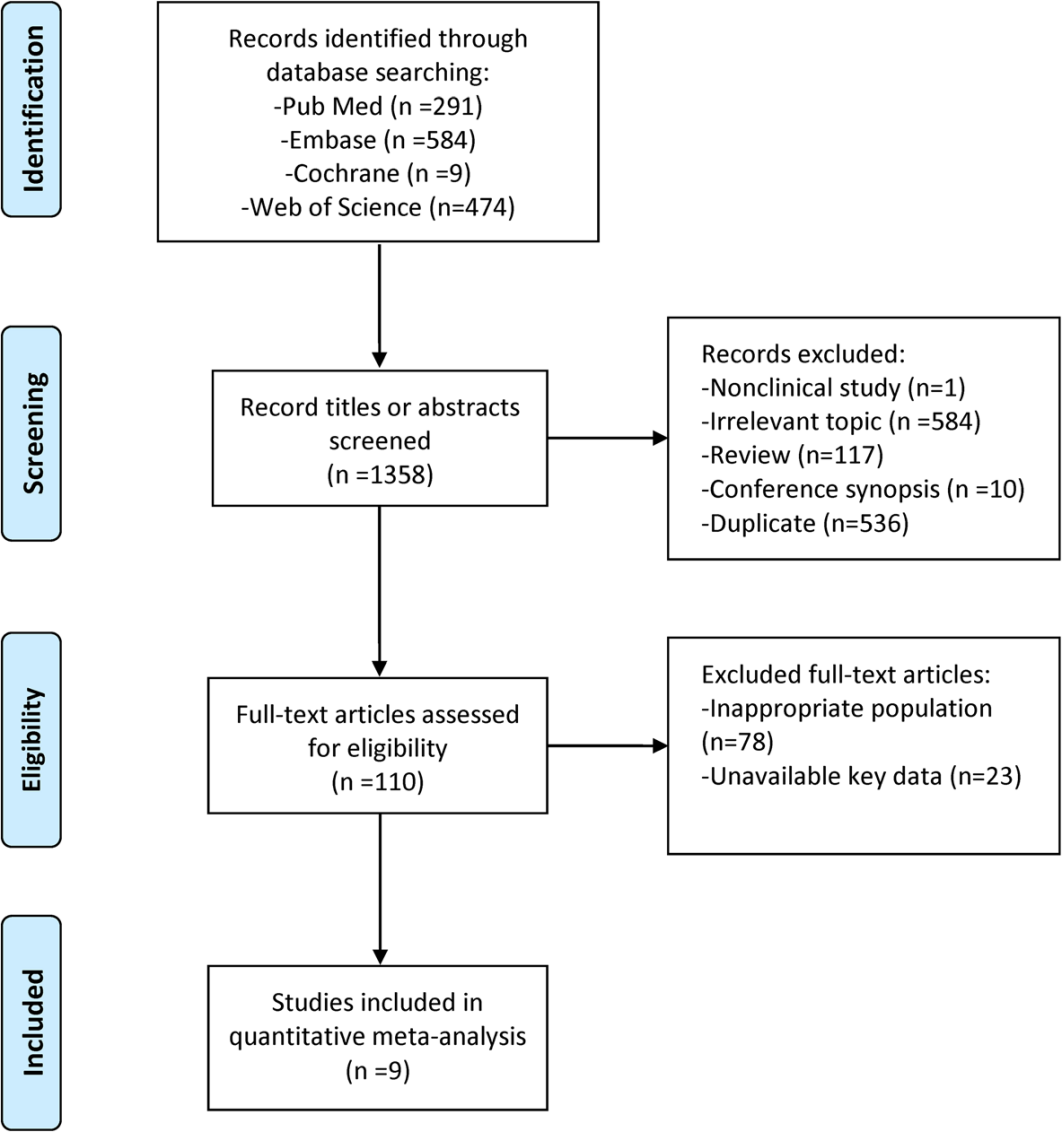
Flow diagram of the study search and selection process

The electronic databases search yielded 1,358 citations as of December 2017, of which 1,248 citations were excluded for various reasons based on the screening of titles and abstracts, leaving 110 studies assessed for full-text review. We further excluded 78 studies in view of the inappropriate study population or23 studies for unavailable key data. Finally, nine studies [24–32] involving 3,337 subjects met our eligibility criteria and were included in the meta-analyses (Fig. 1).

Only subjects in the ischemic stroke and control groups according to MTHFR C677T variant status were included in the meta-analysis. Four studies [24–27] were conducted among Chinese people. Seven of nine studies obtained 6 stars in the NOS. The main characteristics of the included studies are presented in Table 1.

**Table 1:** Characteristic of studies included in the meta-analysis

First Author/ Year [refs]	Ethnicity /Country	Study type	Mean age at baseline, yrs	Women, %	Group description/ Patients, n	Subjects of CC/CT/TT	Subjects of C-Allele/T-Allele	NOS score
Q.-Q. Lv/2015 [24]	Chinese/Asian	Case-control	69	41.7	Ischemic stroke/199	70/98/31	238/160	6
			67	46.5	Control/241	88/116/37	292/190	
Bao-Sheng Zhou/2014 [25]	Chinese/Asian	Case-control	66	36.3	Ischemic stroke/542	160/270/112	382/590	6
			66	38.6	Control/654	242/308/104	792/516	
Lihui Xiong/2012 [26]	Chinese/Asian	Case-control	69	28.0	Ischemic stroke/89	35/42/12	112/66	6
			68	27.0	Control/102	46/45/11	137/67	
Zhang Yan/2008 [27]	Chinese/Asian	Case-control	≥67	NA	Ischemic stroke/105	22/45/38	89/121	6
			≥67	NA	Control/59	18/23/18	59/59	
Ryuichi Kawamoto/2005 [28]	Non-Chinese/Asian	Case-control	78	41.2	Ischemic stroke/97	33/43/21	109/85	6
			76	51.0	Control/241	91/110/40	292/190	
Jun-Hyun Yoo/2000 [29]	Non-Chinese/Asian	Case-control	74	61.0	Ischemic stroke/122	41/69/12	151/93	6
			72	60.0	Control/217	77/114/26	268/280	
Richard D.Press/1999 [30]	Non-Chinese/Non-Asian	Case-control	65	10.0	Ischemic stroke/136	56/71/9	183/89	5
			67	42.0	Control/52	28/23/1	79/25	
Dawn L. Harmon/1999 [31]	Non-Chinese/Non-Asian	Case-control	79	52.9	Ischemic stroke/174	74/73/27	221/127	5
			73	74.9	Control/183	86/78/19	250/116	
K.Kostulas/1998 [32]	Non-Chinese/Non-Asian	Case-control	> 65	NA	Ischemic stroke/62	34/22/6	90/34	6
			> 65	NA	Control/62	25/30/7	80/44	

NA, not applicable; NOS, Newcastle-Ottawa Scale

### MTHFR and ischemic stroke

**Fig. 2:**
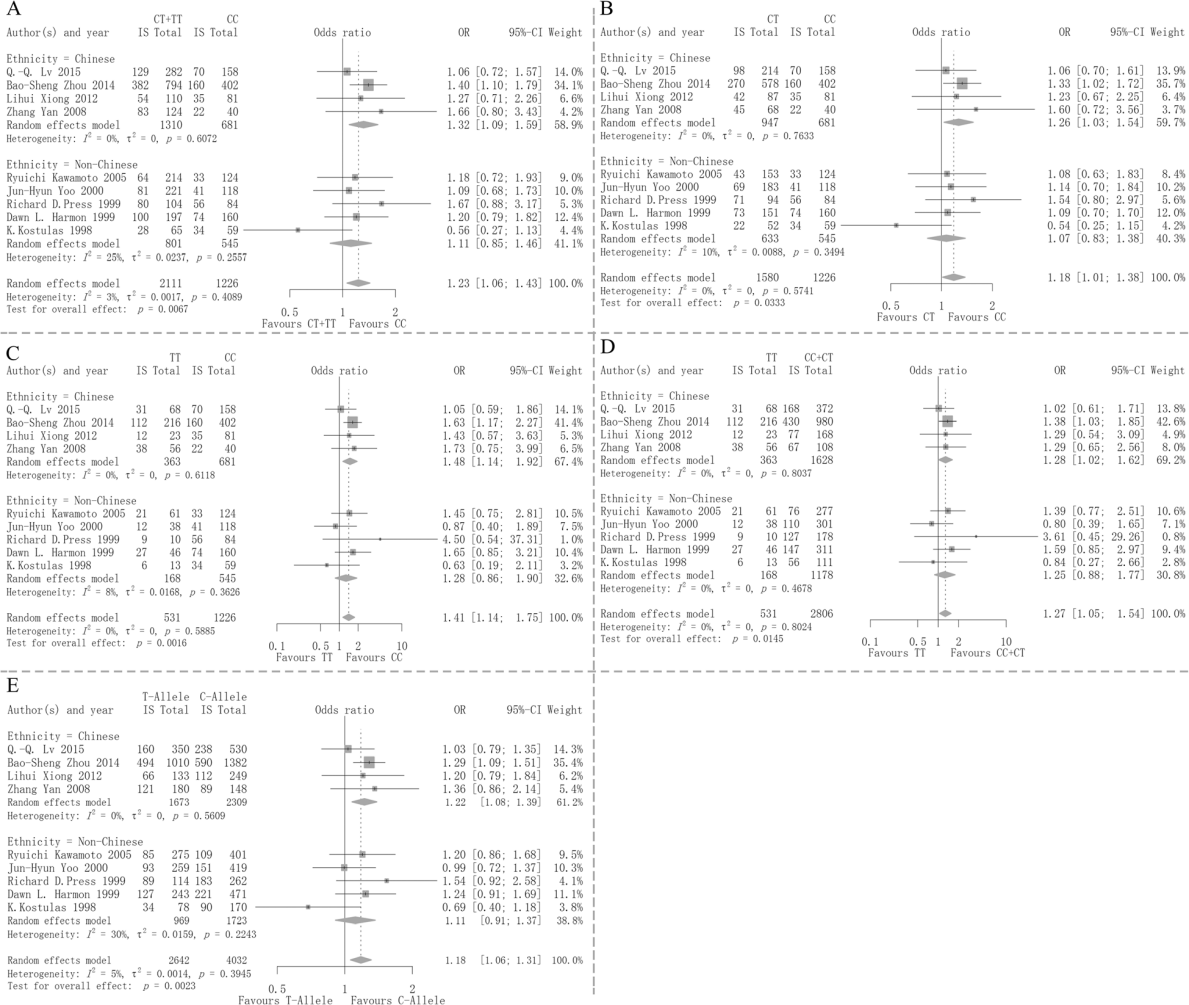
Forest plots of the relationship between MTHFR 677CT polymorphism and ischemic stroke (**a**: CT + TT vs. CC; **b**: CT vs. CC; **c**: TT vs. CC; **d**: TT vs. CC + CT; **e**:T-allele vs. C-allele). CI, confidence interval; IS, ischemic stroke

The pooled results of nine studies showed that MTHFR C677T variant and T-allele expression increased the risk of ischemic stroke remarkably consistently as follows: CT + TT versus CC, OR 1.23, 95%CI 1.06–1.43, *P* = 0.0067, Fig. 2a; CT versus CC, OR 1.18, 95%CI 1.01–1.38, *P* = 0.0333, Fig. 2b; TT vs. CC: OR 1.41, 95%CI 1.14–1.75, *P* = 0.0016, Fig. 2c; TT vs. CC + CT: OR 1.27, 95%CI 1.05–1.54, *P* = 0.0145, Fig. 2d; T-allele versus C-allele: OR 1.18, 95%CI 1.06–1.31, *P* = 0.0023, Fig. 2e.

A further pre-specified subgroup analysis was conducted in the Chinese (*n* = 1,991) and non-Chinese populations (*n* = 1,346). The subgroup results in the Chinese population similarly showed that MTHFR C677T variant and T-allele expression were significantly associated with elevated risk of ischemic stroke as follows: CT + TT versus CC, OR 1.32, 95%CI 1.09–1.59, Fig. 2a; CT vs. CC, OR 1.26, 95%CI 1.03–1.54, Fig. 2b; TT versus CC, OR 1.48, 95%CI 1.14–1.92, Fig. 2c; TT versus CC + CT: OR 1.28, 95%CI 1.02–1.62, Fig. 2d; T-allele vs. C-allele: OR 1.22, 95%CI 1.08–1.39, Fig. 2e. The non-Chinese group did not show a difference and the trend is very similar with its results as follows: CT + TT versus CC, OR 1.11, 95%CI 0,85–1.46, Fig. 2a; CT vs. CC, OR 1.07, 95%CI 0.83–1.38, Fig. 2b; TT versus CC, OR 1.28, 95%CI 0.86–1.90, Fig. 2c; TT versus CC + CT: OR 1.25, 95%CI 0.88–1.77, Fig. 2d; T-allele vs. C-allele: OR 1.11, 95%CI 0.91–1.37, Fig. 2e, indicating that each pooled 95% confidence interval does not contain unity 1.

### Publication bias

**Fig 3:**
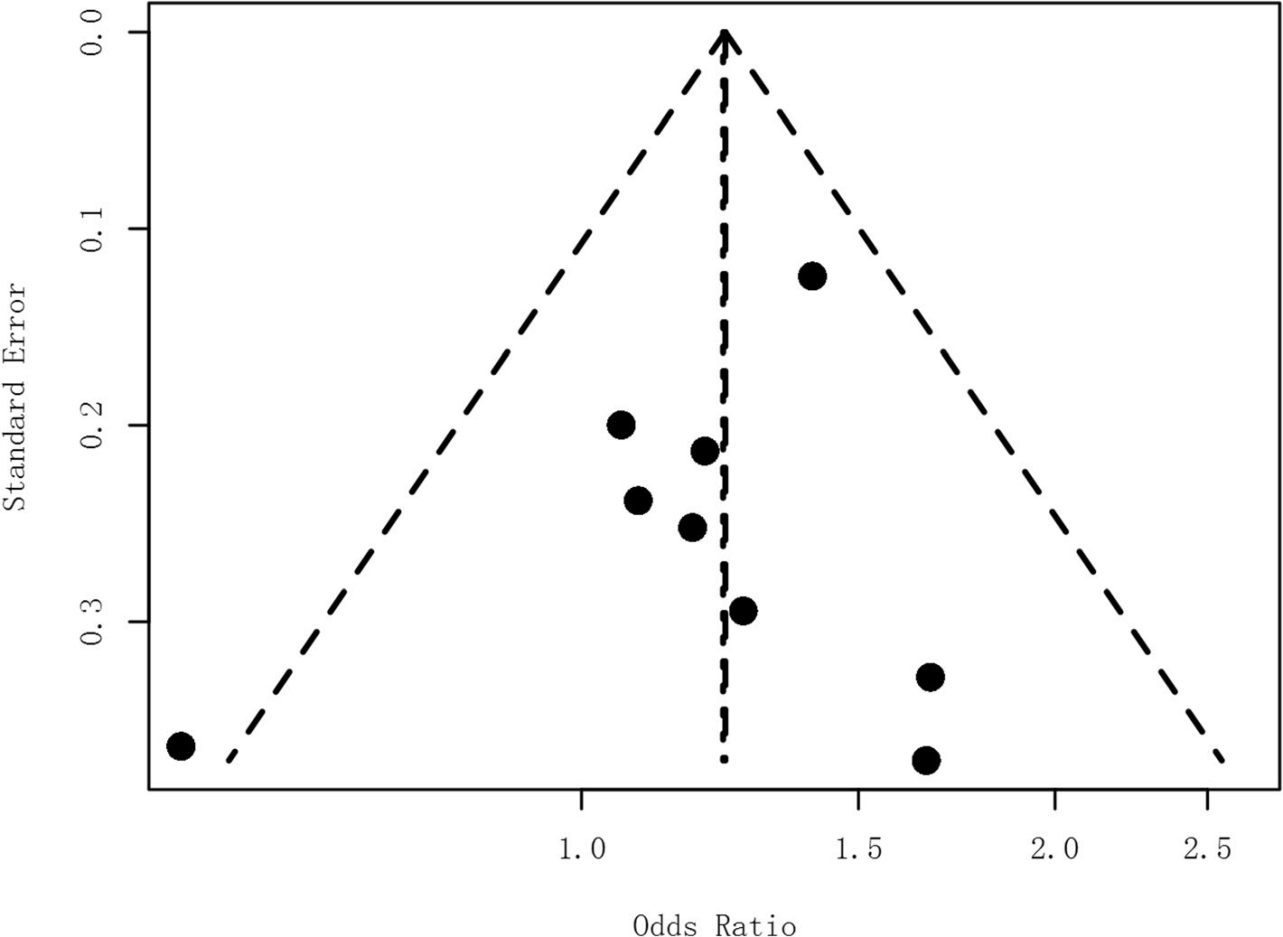
Funnel plot of the-meta-analysis. Funnel plot of the odd ratios against their standard errors (Egger’s linear regression test: *P* = 0.402)

The funnel plots did not show any evidence of obvious asymmetry (Fig. 3). We further used the linear regression method to quantitatively evaluate the symmetry of the funnel plot and found no significant publication bias (*P* = 0.402). We performed a meta-regression analysis including ethnic group as a factor and didn’t found any significant difference among five panels compared (data not shown).

## Discussion

To the best of our knowledge, this study is the first meta-analysis to focus on a specific elderly population to assess the risk of ischemic stroke and MTHFR C677T variant. The large-sample pooled analysis of nine homogeneous case-control studies demonstrated an association between MTHFR C677T variant and increased risk of ischemic stroke as follows: a 23% (95% CI 6–43%) higher risk of ischemic stroke was observed in the MTHFR C677T mutation group than in the control group with no mutation, and up to 32% (95% CI 9–59%) higher risk was observed specifically in the Chinese subgroup.

Hyperhomocysteinaemia is emerging as a potential risk factor of cardiovascular diseases, including cerebral stroke, and is considered modifiable. Both genetics and environmental factors (e.g., dietary intake of folic acid and B vitamins) affect homocysteine level [34]. As one of the most extensively studied gene variants, MTHFR C677T was shown to contribute to ischemic stroke susceptibility, but the results seem inconsistent [35]. In one genetic meta-analysis of 111 studies conducted by Casas JP et.al, individuals homozygous for the T allele of the MTHFR C677T variant have higher plasma homocysteine concentrations than those with the CC genotype [36]. the TT genotype associated with a predisposition to increased plasma homocysteine levels may represent a genetic risk factor for CAD [37]. However, in recent years, contradictory results have also been reported. Pramukarso DT et al. observed 71 patients with post-ischemic stroke and found that the MTHFR 677T allele polymorphism is not associated with hyperhomocysteinemia [38]. Hyperhomocysteinemia has been associated with mutations of the MTHFR gene, mainly the C677T and A1298C mutations. A. A. D. Gayathri UpekshaAmarakoon et al. mentioned that a conclusive result was given, as the C677T genotype in its homozygous wild type condition, renders the individual at no risk, regardless of the genetic condition at the 1298 position. Similarly, the homozygous mutant of C677T renders the individual at risk and does not depend on the 1298 position genotype [39]. C677T variant is generally accepted to contribute to decreased enzyme activity and ultimately confers increased susceptibility to ischemic stroke. C677T variant is generally accepted to contribute to decreased enzyme activity and ultimately confers increased susceptibility to ischemic stroke. This meta-study is consistent with previous study of meta-analysis [40], which might support that the MTHFR C677T mutation is likely to be a direct genetic risk factor of ischemic stroke [33] and TT genotype would become a promising biomarker for the early detection and prediction of the prognosis of ischemic stroke as well as regarding to safely conducting future prospects of research [39] in the elderly population.

In China, with a population of 1.4 billion, the annual stroke mortality is approximately 1.6 million, and approximately 43 to 79% of all stroke cases are ischemic [41, 42]. This meta-analysis indicated that the TT genotype had a significant association with increased risk of ischemic stroke in the elderly Chinese population, with an elevated risk of 41% (95% CI 14–75%), higher than that for CC genotype. This may be related to the following arguments [43, 44]: (1) Chinese people appear more susceptibleto MTHFR C677T mutation; (2) poor dietary intake of folic acid and vitamin B12, which are related to Chinese dietary habits; and (3) an increased level of homocysteine status, which is an important risk factor of cardiovascular diseases. Adequate folic acid intake appears to reduce the risk of stroke through down-regulation of plasma homocysteine concentration, which was deemed as a strong and independent influencing factor of stroke [45]. A previous study reported that a 25% lower usual homocysteine level was associated with a 19% lower risk of stroke [46]. With these, we might consider that folic acid intake and homocysteine control would be a pragmatic preventive measure for ischemic stroke.

This meta-analysis showed that MTHFR C677T variant may be associated with increased risk of ischemic stroke in the elderly, specifically a significant difference among the Chinese population and a similar trend among the non-Chinese population as the non-Chinese subset is smaller than the Chinese subset which can be explained by a difference in power. However, we agree that this meta-analysis has several potential limitations. First, the study was based on secondary study-level data. All the included studies in our meta-analysis met the required mean (median) age of ≥65 years for study subjects, but this is not to say that each subject from the included studies was aged > 65 years. Second, the homocysteine concentration was measured only in two of the included studies [29, 30], which makes it impossible for us to analyse it as an independent factor in this meta-analysis. Third, A1298C is a second common variant of MTHFR which clearly reduces MTHFR activity, albeit to a lesser extent than the C677T mutation, which effect on homocysteine levels is also attenuated and may only be significant when an individual carries both mutations and/or has poor nutrient status [47]. The association between MTHFR A1298C variant and the risk of ischemic stroke was investigated only in two of the included studies [24, 25], which makes it impossible for us to analyse it as an independent factor in this meta-analysis. Moreover, some fluctuations of population characteristics were observed, which could be a major source of heterogeneity, although the analysis of heterogeneity did not show any significance. Nonetheless, the findings of this meta-analysis which were based on case-control studies alone, should be interpreted with caution.

## Conclusions

In conclusion, our findings showed that MTHFR C677T variant may contribute to increased risk of ischemic stroke in the elderly population; T-allele increases risk for stroke in the pooled sample. This association was statistically significant in the Chinese cohorts and showed a similar trend in the non-Chinese cohorts. For elderly people, MTHFR C677T might be a promising biomarker for the early detection and prediction of the prognosis of ischemic stroke. For more solid evidence, however, well-controlled prospective studies are warranted in the future.

## Data Availability

All the data supporting our findings of this study are available in the reference [24–32].

## Abbreviations

CI: Confidence interval
MTHFR: Methylenetetrahydrofolate reductase
NA: Not applicable
NOS: Newcastle-Ottawa Scale
OR: Odds ratio
SNP: Single nucleotide polymorphism
